# Neural Basis of Extremely High Temporal Sensitivity: Insights From a Patient With Autism

**DOI:** 10.3389/fnins.2020.00340

**Published:** 2020-04-30

**Authors:** Masakazu Ide, Takeshi Atsumi, Mrinmoy Chakrabarty, Ayako Yaguchi, Yumi Umesawa, Reiko Fukatsu, Makoto Wada

**Affiliations:** ^1^Department of Rehabilitation for Brain Functions, Research Institute of National Rehabilitation Center for Persons with Disabilities, Saitama, Japan; ^2^Japan Society for the Promotion of Science, Tokyo, Japan; ^3^Department of Medical Physiology, Faculty of Medicine, Kyorin University, Tokyo, Japan; ^4^Department of Social Sciences and Humanities, Indraprastha Institute of Information Technology (IIIT-D), New Delhi, India; ^5^Department of Contemporary Psychology, Rikkyo University, Saitama, Japan

**Keywords:** temporal resolution, temporal order judgment, autism spectrum disorder, functional magnetic resonance imaging, inferior frontal gyrus

## Abstract

The human brain is sensitive to incoming sensory information across multiple time scales. Temporal scales of information represented in the brain generally constrain behavior. Despite reports of the neural correlates of millisecond timing, how the human brain processes sensory stimuli in the sub-second range (≤100 ms) and its behavioral implications are areas of active scientific inquiry. An autism spectrum disorder (ASD) patient showed a tactile discrimination threshold of 6.49 ms on a temporal order judgment (TOJ) task which was approximately 10-fold superior than other ASD and healthy controls (59 and 69 ms, respectively). To investigate the brain regions of this extremely high temporal resolution in the patient, we used functional magnetic resonance imaging (fMRI) during TOJ. We observed greater activity notably in the left superior temporal gyrus (STG) and precentral gyrus (PrG) compared to that of controls. Generally, the left superior frontal gyrus (SFG) correlated positively, while the opercular part of right inferior frontal gyrus (IFG) correlated negatively, with the correct TOJ rate across all subjects (the patient + 22 healthy controls). We found that the performance was negatively correlated with the strength of neural responses in the right IFG overall in 30 participants (the patient + 22 healthy and 7 ASD controls). Our data reveal superior ability of this particular case of ASD in the millisecond scale for sensory inputs. We highlight several neural correlates of TOJ underlying the facilitation and/or inhibition of temporal resolution in humans.

## Introduction

To engage with the external environment, humans process temporal information across a wide range of intervals. Various distributed neural substrates process temporal information with varying degrees of resolution and precision based on specific task demands (Buhusi and Meck, 2005). Neural correlates of millisecond timing over several tens to hundreds of milliseconds in healthy subjects have revealed brain regions functionally related to temporal order judgment (TOJ) (Davis et al., 2009; Takahashi et al., 2013; Binder, 2015; Miyazaki et al., 2016; Takahashi and Kitazawa, 2017), where participants judge the order of first/last of two successive stimuli with varying stimulus onset asynchronies (SOAs). However, whether these brain areas are also involved in resolving extremely fine sub-second interval timing is open to question.

Superior local processing abilities of stimulus elements reported in autism spectrum disorder (ASD) (Dakin and Frith, 2005) are considered an overdevelopment of low-level perceptual abilities that enhance detection and discrimination of stimuli. For example, a superiority in visual search tasks demanding detection of targets with different features defined by color, shape, or those combinations from surrounding distractors have frequently been reported (O’Riordan et al., 2001; O’Riordan, 2004; Kaldy et al., 2016; Cheung et al., 2018). However, little is known about their superior processing of sensory inputs. A study showed that ASD had better temporal resolution of vibrotactile stimuli delivered with a synchronized 25-Hz vibrotactile stimuli than typically developing (TD) peers (Tommerdahl et al., 2008). We, however, found no significant difference in tactile temporal resolution (40-Hz and 200-Hz vibrotactile stimuli) between ASD and TD, although individual differences of temporal resolution were associated with the severity of sensory hyper-responsivity (Ide et al., 2019).

Miyazaki et al. (2016) suggested that the left ventral premotor cortex (vPMC), bilateral dorsal premotor cortex (dPMC), and the left posterior parietal cortex (PPC) extending to the marginal area contiguous to the left superior temporal gyrus (STG) of the motor control network (Kurata, 1993; Pisella et al., 2000) were strongly activated during TOJ task in comparison with a simultaneity judgment task. Since the TOJ-specific cerebral activations were primarily lateralized to the left hemisphere (Miyazaki et al., 2016), neural activation in the left side of the brain associated with motor control could be assumed to be the neural correlates of sub-second temporal resolution.

Another comparison with number judgment task suggested that the TOJ-related regions include the bilateral ventral frontal cortices (VFCs) (Takahashi et al., 2013). A study elucidated the brain activities of TOJ in three difficulty conditions of SOA (easy: 160 ms, moderate: 60 ms, difficult: 10 ms) (Lewandowska et al., 2010). Results of the regression analysis demonstrated significant positive relationships between task difficulty and blood oxygen level-dependent (BOLD) responses in the bilateral inferior frontal gyrus (IFG). Right IFG, one of the brain regions reported to activate during TOJ (Davis et al., 2009; Lewandowska et al., 2010; Takahashi et al., 2013; Binder, 2015), has been suggested to associate with response inhibition to stop-signals (Aron et al., 2003; Depue et al., 2010), especially for tactile stimuli, compared to visual or visuotactile modalities (Bodmer and Beste, 2017). From these previous studies, the right IFG would be a substantial brain area for producing high temporal resolution by modulating somatosensory inputs, especially when intervals of the stimulus presentation are very short. However, the way in which individual sub-second temporal resolution is modulated has been unknown, to our knowledge.

We found a particular ASD patient (T.R.) who showed extraordinary ability to resolve sub-second timings in tactile TOJ (bilateral hands) compared to TD and other ASD participants. We first examined if this superior ability was shared with other sensory modalities: auditory, visual, and unilateral, one-hand tactile. Next, we measured T.R.’s neural activity during bilateral tactile-TOJ of hands to elucidate neural correlates of this exceptional temporal processing, focusing on brain regions which facilitate and/or inhibit brain activity arising by tactile temporal processing. Finally, we tested if the brain regions contribute to the modulation of temporal resolution by a regression with general individual performance levels of TOJ across TD and ASD participants.

Regions of interest (ROIs) in the present study were hypothesized based on the following earlier studies that reported brain activity preferentially for the TOJ task. The left vPMC and STG (Adhikari et al., 2013; Takahashi et al., 2013; Miyazaki et al., 2016) were found to contribute to accurate TOJ of tactile stimuli. In the mask applied for small volume correction (SVC) analyses in the current study, vPMC is an anatomically overlapping part of precentral gyrus (PrG) (see more details in the “Materials and Methods” section). The left superior frontal gyrus (SFG), reportedly a TOJ-related region (Takahashi et al., 2013; Binder, 2015) also serves working memory (WM). The SFG was expected to play a crucial role in governing temporal resolution since WM functional capacity together with increased brain activity in the bilateral frontal cortices has been suggested to be important for performing TOJ (McAndrews and Milner, 1991; Soto et al., 2008; Binder, 2015). Stronger neural activity was observed in the bilateral IFG with increased task difficulty during TOJ (Lewandowska et al., 2010). Especially the right IFG is known for response inhibition (Aron et al., 2003; Depue et al., 2010) and interference suppression (Konishi et al., 1999). Thus, it was another candidate area of inhibitory control of temporal resolution. Accordingly, the left PrG which is the anatomically corresponding part of vPMC, SFG, STG, and the right IFG masks were defined as *a priori* ROIs.

## Materials and Methods

### History of the Autism Spectrum Disorder Patient

The ASD patient T.R. (fictitious initials to protect true identity) was a 19-year-old right-handed male. He was clinically diagnosed with pervasive developmental disorders with no intellectual disability at the age of 3 years. He was on atomoxetine hydrochloride every day including the day of experiments. His diagnostic level assessed by Autism Diagnostic Observation Schedule-Second Edition (ADOS-2) (Lord et al., 2012) met the criteria of “autism spectrum” (Table 1). Intelligence Quotients (IQs) evaluated by the Wechsler Adult Intelligence Scale-Third Edition (WAIS-III) indicated that all scores (Full-scale IQ, Verbal IQ, and Performance IQ) including sub-scores (Verbal Comprehension Index, Working Memory Index, Perceptual Organization Index, and Processing Speed Index) fell within 1 standard deviation (SD) of the standardized means.

**TABLE 1:** Diagnostic information of patient T.R.

		**Scores**	**Severity**
**ADOS-2**	Total*	**7 (Cutoff: 5)**	**Autism spectrum**
	Communication	2	
	Reciprocal social interaction	5	
	Stereotyped behaviors and restricted interests	2	
IQ	FSIQ	96	Average
	VIQ	95	Average
	PIQ	98	Average

*Total score in ADOS-2 is summarized value of Communication and Reciprocal social interaction subscores. ADOS-2, Autism Diagnostic Observation Schedule-Second Edition; IQ, Intelligence Quotient; FSIQ, Full Scale IQ; VIQ, Verbal IQ; PIQ, Performance IQ

Patient T.R. reported a particular liking for the movement of the “second hand” of clocks and would often stop by watch stores to compare the synchronicity of the “second hand” of different watches. T.R. complained of abnormal tactile and auditory sensations in the clinical interview. He stated his predicament with his father’s new car. He initially did not realize why he felt as such, but gradually noticed that slight changes in vibration from car seats caused marked unpleasant sensations. He also complained of heightened sensitivity to sound. He effortlessly noticed technical malfunctions in headsets that led to minor changes in audio quality.

### Control Subjects

In deciding the sample sizes, we referred to several functional magnetic resonance imaging (fMRI) case studies that compared brain activities of a patient in cognitive tasks with healthy control subjects (Mullally et al., 2012; Vo et al., 2014; Claessen et al., 2019; Loosli et al., 2019), and the sample size of the present study was comparable to these earlier studies. Sixteen healthy subjects as TD controls (seven females; mean age 23 years ± 4 SD) and 15 patients with ASD (other than T.R.; two females; mean age 20 years ± 4 SD) also performed bilateral tactile TOJ task as controls. All patients were clinically diagnosed as ASD and were recruited from either parent groups for children with developmental disorders or the Hospital of National Rehabilitation Center for Persons with Disabilities.

Temporal resolution was tested for different TOJ modalities in TD control subjects and compared with T.R.: 16 controls (six females; mean age 23 years ± 6 SD) for bilateral auditory, 15 controls (seven females; mean age 24 years ± 6 SD) for binocular visual, and 16 controls (six females; mean age 25 years ± 6 SD) for unilateral right hand tactile. Supplementary Table 1 denotes the overlap of TD controls across different modalities of the TOJ task.

The fMRI experiment was performed firstly by TD controls for bilateral tactile TOJ and numerosity judgment (NJ): 22 TD controls (10 females; mean age 23 years ± 5 SD). fMRI experiments with two control subjects failed due to malfunctioning of the Braille stimulator during scanning (i.e. 24 controls initially), and their data were excluded from final analyses (24–2 = 22 controls for the analysis). Brain images of seven control ASD subjects (two females; mean age 21 years ± 3 SD) with 22 TD controls and T.R. (i.e. *N* = 30 in total) were used for multiple regression analysis. The structural image of one control ASD patient could not be acquired, which we repeated on a separate day.

All participants and their parents gave written informed consent according to the Declaration of Helsinki after the study procedures had been fully explained. The present study was approved by Ethics committee of the National Rehabilitation Center for Persons with Disabilities.

### Behavioral Experiments

Vibrotactile stimuli were generated with varying temporal lags; ±15, 30, 60, 120, 240 ms. The trials with negative SOAs indicate that the right stimulator worked earlier than the left one. Initially, we could not correctly measure T.R.’s performances since his temporal resolution was superior to the minimum cutoff SOA of control subjects; that is, he perfectly performed tactile TOJ with the shortest SOA of 15 ms when 40 and 200 Hz vibrations were delivered in the practical test (see Supplementary Figure 1). In this case, the calculated temporal resolution did not reflect actual performance since it would be less than 15 ms. Thus, a special set of SOA conditions, ±1, 5, 10, 15, 30, 60, 120, 240 ms, were adopted for T.R. in the TOJ tasks across tactile (bilateral hands and one hand), auditory, and visual modalities.

#### Bilateral Tactile Temporal Order Judgment

Solenoid skin contactors (FR-2007-2α, Uchida Denshi, Tokyo, Japan) were used to deliver vibrotactile stimulation to both hands. The displacement (2 μm) and duration (1 ms) of the vibrations were measured by a laser displacement meter (LK-G15, KEYENCE, Osaka, Japan). White noise (54.7 dB) was delivered through headphones (HD380PRO, SENNHEISER, Wedemark, Germany) to prevent ambient noise from affecting the task performance. The stimulators were controlled by a custom script written in MATLAB (R2007b, MathWorks, Natick, MA, United States) run on Dell precision workstation (TS5500, Dell, Japan).

Two vibrotactile stimuli were successively delivered to both left and right index fingers with various temporal lags. The set of SOAs was exclusive for T.R. due to his extraordinarily high temporal resolution as described above, whereas SOAs of ± 15, 30, 60, 120, and 240 ms were used for control subjects. The trials with negative SOAs indicated that the right stimulator worked earlier than the left one.

Subjects placed their left and right fingers on the response buttons of the respective sides. Subjects were required to respond on the side on which the latter stimulus was delivered by pressing the response button of that particular side as quickly as possible. Each SOA condition randomly appeared 10 times in one block, and the block was repeated twice (20 times in total for each SOA) with approximately 5 min rest period. Subjects closed their eyes throughout the experiment.

#### Auditory Temporal Order Judgment

Pure tones (500 Hz, 63.5 dB) were delivered through the headphones using a customized sound generator (FSS-002, Uchida Denshi, Tokyo, Japan). White noise was always delivered in the background. Other experimental conditions were the same as those in tactile TOJ.

#### Visual Temporal Order Judgment

Light-emitting diodes (LEDs) (FR-2007-2α, Uchida Denshi, Tokyo, Japan) were used as visual stimuli and flashed on the index and ring fingers. All other experimental conditions were the same as those in tactile TOJ.

#### One-Hand Tactile Temporal Order Judgment

All experimental conditions were the same as those in bilateral tactile TOJ, except that vibrotactile stimuli were delivered to the index and ring fingers of the right hand only.

### Statistics

Temporal resolution was calculated by fitting the response data in each task to a Gaussian cumulative density function (Yamamoto and Kitazawa, 2001; Ide et al., 2019). The response data were sorted by the SOAs to calculate the order-judgment probability that the right index finger was stimulated later (or the left index finger was stimulated first). Data with reaction times longer than 3,000 ms were excluded from the analysis. The judgment probabilities of the data in the TOJ task were fitted using the following function:

$$p\,\left(t\right)=\left(p_{m\,a\,x}-p_{m\,i\,n}\right)\,\int_{-\infty }^{t}\frac{1}{\sqrt{2\,\text{ π }\,\sigma_{t}}}\,e\,x\,p\,\left(\frac{-{\left(\tau -d_{d}\right)}^{2}}{2\,\sigma_{d}^{2}}\right)\,dt\,\tau +p_{m\,i\,n}$$

where *t*, *d*, *σ*, *p*_*max*_, and *p*_*min*_ represent the SOAs, size of the horizontal transition, temporal resolution, and upper and lower asymptotes of the judgment probability, respectively. The *σ* corresponded to the stimulation interval that yielded 84% correct responses (relative to the asymptote). The MATLAB (R2015a) Optimization toolbox was used to minimize the Pearson’s chi-square statistic in the model fitting, which reflects the discrepancy between the sampled order-judgment probability and the prediction using the four-parameter model.

We used the Bayesian approach for single-case studies (Crawford and Garthwaite, 2007) which is advantageous because the change of sample size of controls does not greatly affect the statistical results. The Bayesian standardized difference test (Crawford and Garthwaite, 2007) has been developed to compare one sample with controls, reducing the probability of Type I error when a patient shows extremely low (or high) score in some tasks (Crawford and Garthwaite, 2007). The computer program “SingleBayes_ES.exe” was used with 95% confidence interval (CI) setting “one-sided lower.”

### Task-Related Functional MRI

Tactile TOJ and control NJ (Takahashi et al., 2013) tasks were used to identify the brain areas specifically activated during the temporal processing of stimuli. A block design was used for these two fMRI tasks. We employed a non-magnetic Braille stimulator with eight movable pins, manufactured by KGS Corporation (Saitama, Japan) following the published design (Takahashi et al., 2013). The stimulator consisted of a response button on the roof of which eight pins were aligned as a 4 × 2 array with inter-pin intervals of 3 mm. The pins were embedded inside the button, which were pushed up by the piezoelectric actuators delivering a vibrotactile stimulus to the fingers of the subjects. The piezoelectric actuators moved the pins mounted on left and right stimulators once per trial. The timing of stimulation and the number of pins pushed up during each stimulation (depending on the experimental condition) were controlled by a custom written program in our lab on Python for MacBook Pro (Apple, Inc., Japan).

Two stimulators delivered the stimuli with various temporal lags during every trial. We separated SOAs into two conditions (i.e. Short SOA and Long SOA): Short SOA condition contained intervals of ± 1, 5, 10, and 15 ms, while Long SOA condition involved intervals of ± 25, 50, 75, and 100 ms. This was to maintain the ASD patient’s arousal to the task by including rest periods at adequate intervals, as it was sometimes difficult for the patient to continue the task for a long time without separating blocks. Trials with negative SOA indicate that the right stimulator worked earlier than the left did. In every trial, one out of two stimuli delivered the stimulus with one pin more than the other. In total, there were four such combinations: 8 versus 7, 7 versus 6, 6 versus 5, and 5 versus 4 pins. Each combination appeared twice, with sides flipped once. The order of SOAs and pin number combinations were pseudorandomized across trials.

Subjects held the two Braille stimulators in each hand and placed the ventral side of their index fingers on each response button during fMRI scanning. To isolate the brain activations that occurred specifically during the TOJ task, the NJ task was employed as a control. In the TOJ task, subjects answered which of the left or right stimulators delivered the latter stimuli by pressing the corresponding response button. In the NJ task, the subjects answered the side on which the stimulator delivered a greater number of pins by pressing the corresponding button.

Each subject underwent two fMRI sessions, one with the TOJ task and the other with the NJ task. Each session comprised eight blocks; four out of eight blocks were assigned to Task blocks while the remaining were Rest blocks. Each session started with a Task block and thereafter alternated with Rest blocks. A block lasted for 48.1 s, and subjects performed eight trials with all SOA and pin combinations. Each trial started after an intertrial interval (ITI) of 2 s, and then two successive stimuli were delivered with various delays. Soon after the presentation of stimuli, the subjects were required to press the response button within 3 s. After the passage of response time, ITI started immediately. Two types of task blocks (Long SOA and Short SOA) appeared sequentially within a session. The order of task blocks and session types (TOJ and NJ) were counterbalanced across the controls.

T.R. started with the TOJ task session. To confirm the effect of the session order on neural activation, he was scanned again with the task sessions in reverse order 83 days after the initial scanning.

### MRI Acquisition

fMRI data were acquired on a 3T Siemens Skyra scanner (Siemens, Erlangen, Germany) with 64 channel head coil. Functional images sensitive to the BOLD contrast (Ogawa et al., 1993) were obtained from a T2^∗^ gradient-echo planar imaging (EPI) pulse sequence [TR = 2,620 ms; TE = 30.0 ms; flip angle = 80°; field of view (FoV) = 281 mm; voxel size = 2.2 × 2.2 × 3.2 mm^3^; slice thickness = 3.2 mm; slice number = 39; interslice gap = 1.28 mm]. A total of 146 volumes were acquired in an experimental duration of 6.4 min separately for TOJ and NJ tasks. A high-resolution T1-weighted anatomical image was acquired using a magnetization-prepared rapid acquisition by gradient echo sequence (TR = 2,300 ms; TE = 2.98 ms; flip angle = 120°; FoV = 256 mm; voxel size = 1.0 × 1.0 × 1.0 mm^3^).

### Functional MRI Analyses

fMRI images were preprocessed and analyzed using SPM12 ver. 7219^1^ and implemented with MATLAB (R2015a). Briefly, the functional images of each experimental session (i.e. run) were realigned, slice time was adjusted; mean functional image of each session was coregistered to the structural image, spatially normalized to standard T1-template image defined by the Montreal Neurological Institute (MNI), and spatially smoothed with a Gaussian kernel of 8-mm full-width at half-maximum.

The preprocessed data from each subject was first entered into fixed effect analysis where task-related neural activity relative to baseline was modeled using a boxcar function, convolved using a canonical hemodynamic response function and filtered by the high-pass filter with a cutoff period of 128 s to rule out low-frequency trends. The statistical model consisted of TOJ and NJ conditions. T-contrast was defined for the TOJ > NJ comparison. The six head motion parameters estimated earlier were regressed out as covariates of no interest in the contrast. Images of the T-contrast were moved into the second-level random effect analyses for isolating activations using Crawford’s modified two-sample *t* test (Crawford and Howell, 1998; Crawford et al., 2006) between the patient and controls. For relating the TOJ brain activations obtained (TOJ > NJ first-level contrasts) with subject wise behavioral scores [tactile TOJ (% correct)], correlational analyses were performed at second level.

Anatomical masks were defined using Wake Forest University PickAtlas Toolbox (Maldjian et al., 2003; Maldjian et al., 2004) and its embedded Automated Anatomical Labeling (AAL; Tzourio-Mazoyer et al., 2002). The ROIs were chosen based on several studies that reported brain areas preferentially for the TOJ task (see the “Introduction” section). The anatomical masks were then used in SVC analyses as explained next. Voxels reported significant in all group level results were those that survived an initial height threshold of *p* < 0.001, uncorrected at voxel level (*Z* > 3.09) to isolate clusters, and *p* < 0.05, Family Wise Error (FWE)-corrected for multiple comparisons at the cluster level (within the small volume of the *a priori* ROIs). An exception to *p*FWE < 0.05 correction at cluster level was for group-level activation (TOJ > NJ) in the left SFG (*p*FWE = 0.08; see the section “Results” for explanation). For labeling the brain areas, SPM Anatomy toolbox (Eickhoff et al., 2005) was used. Areas not reported by this source were identified by MRIcron^2^. Brodmann areas (BAs) were labeled using mni2tal^3^ which is part of the Yale BioImage Suite image analysis package (Lacadie et al., 2008). Remaining unidentified areas (BAs) were labeled using Talairach Client (ver. 2.4.3^4^) after transforming the MNI coordinates of the peak activations into Talairach space using mni2tal.m, an extension program for coordinate transformation^5^.

## Results

### Behavioral Experiments

**FIGURE 1:**
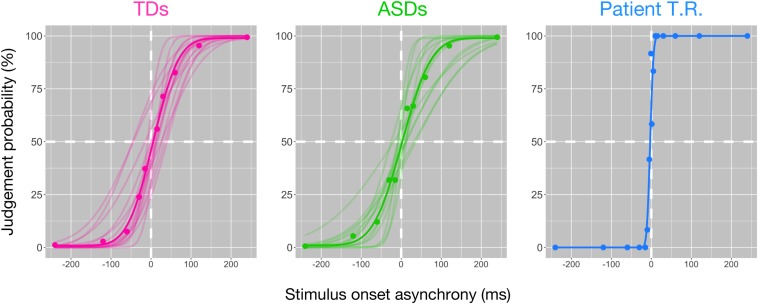
Behavioral findings of the bilateral tactile temporal order judgment (TOJ) task in patient T.R. and control subjects. The judgment probability (*y*-axis) that stimuli delivered to one side were earlier than the other is plotted against the stimulus onset asynchrony (*x*-axis). Positive values along *x*-axis indicate that the right side was stimulated first. Each filled circle represents judgment probabilities calculated from 20 responses for the patient and those of average values for each control group. Curve represents the model fit to the data; thin curves indicate individual functions, and thick curves denote the average of all subjects (see section “Materials and Methods”) [typically developing (TD) control (magenta); autism spectrum disorder (ASD)-control (green)] and T.R. (blue).

We performed tactile TOJ to psychophysically test for differences between controls (TD and ASD) and T.R. in the ability to discriminate two successive temporally spaced tactile vibrations to either hand. Tactile stimuli were generated with varying temporal lags for the task. The temporal resolution was 58.57 ± 21.32 ms (mean ± SD) and 68.74 ± 36.45 ms (mean ± SD) in TD and ASD controls (Figure 1; lower values indicate better temporal resolution), respectively, and there was no significant difference between them [*t* (20.36) = 0.92; Cohen’s *d* = 0.35; 95% CI -33.30, 12.97]. Bayesian standardized difference test (Crawford and Garthwaite, 2007) demonstrated that while T.R.’s temporal resolution (6.49 ms) was significantly greater than TD controls (*z* = −2.44; *p* = 0.015; 95% CI 0.00, 0.06), it was marginally greater than ASD controls (*z* = −1.71; *p* = 0.062; 95% CI 0.00, 0.16).

**FIGURE 2:**
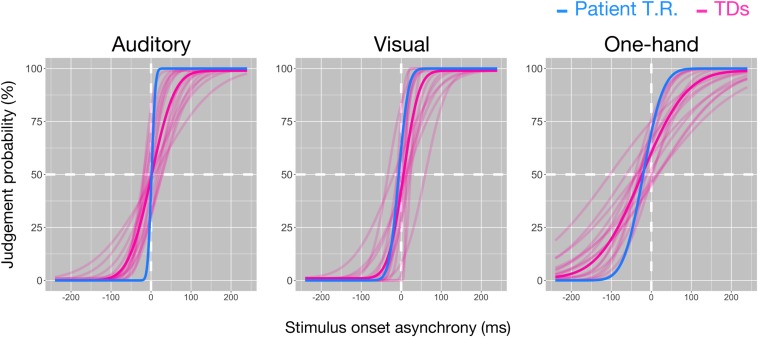
Temporal resolutions in the temporal order judgment (TOJ) task in patient T.R. and control subjects. The judgment probability (*y*-axis) that stimuli (left: auditory, center: visual, right: one-hand tactile) delivered to one side (auditory and visual: left/right lateral, tactile: left [index finger]/right [ring finger] of right hand) were earlier than the other is plotted against the stimulus onset asynchrony (*x*-axis). Positive values in *x*-axis indicate that the right side was stimulated first. Curves resulting from model fit (see “Materials and Methods” section) are shown for the patient (blue) and controls (magenta), respectively, (auditory: *N* = 16, visual: *N* = 15, one-hand tactile: *N* = 16). Thick magenta curves denote the average of all typically developing (TD) subjects.

Temporal resolution (7.42 ms) in T.R.’s discrimination of pure auditory tones delivered to both ears was marginally higher than TD controls [48.71 ± 24.97 ms (mean ± SD); *z* = −1.65; *p* = 0.064; 95% CI 0.00, 0.16] (Figure 2). However, his temporal resolution for visual stimuli (19.09 ms) was comparable to controls [34.3 ± 22.9 ms (mean ± SD); *z* = −0.66; *p* = 0.262; 95% CI 0.11, 0.45], and one-hand tactile TOJ (39.35 ms) was marginally higher than controls [102.59 ± 42.42 ms (mean ± SD); *z* = −1.49; *p* = 0.084; 95% CI 0.00, 0.20]. The results suggested that T.R.’s superior temporal resolution in the bilateral tactile, but not auditory, visual, and one-hand tactile TOJ.

### Task-Related Functional MRI

**FIGURE 3:**
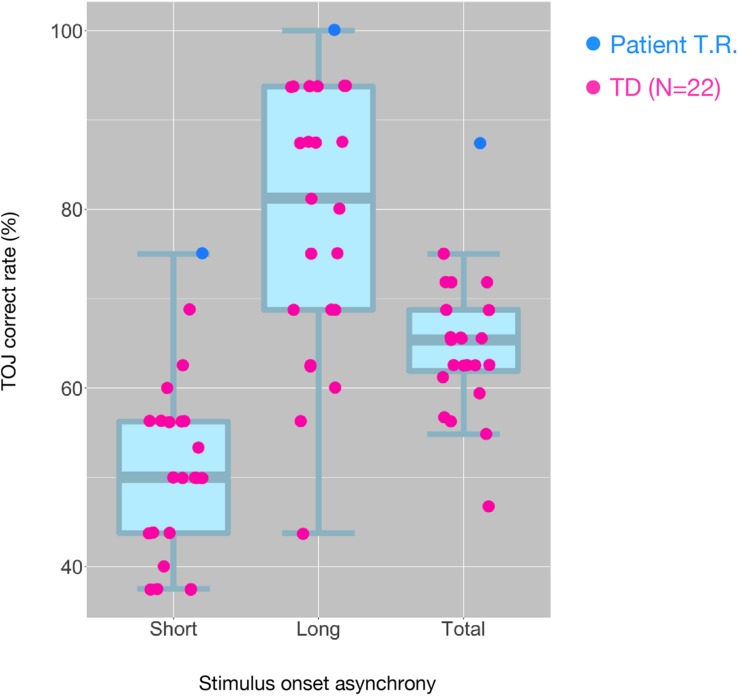
Distribution of tactile temporal order judgment (TOJ) performance of all subjects inside the MRI scanner. The box and whisker plots with the superimposed data points show the distribution of correct TOJ rates of the subjects in the “Short SOA,” “Long SOA,” and “Total (Short SOA + Long SOA)” conditions, respectively. Each box and whisker plot show the 10th, 25th, 50th, 75th, and 90th percentiles of the data. Each filled circle represents correct rate calculated from 32 trials in patient T.R. (blue) and TD controls (magenta). SOA, stimulus onset asynchrony.

T.R.’s correct response rates in TOJ within the MRI scanner were appreciably greater than those of TD controls (100% versus 77.96% and 75% versus 49.87% in the Long SOA and Short SOA conditions, respectively; Figure 3). We performed Bayesian standardized difference test (Crawford and Garthwaite, 2007) to examine whether T.R.’s overall performance of TOJ inside scanner was greater than the case controls. Results showed significant departure of T.R.’s correct rate from controls [63.80 ± 6.55 % (mean ± SD); *z* = 3.62; *p* < 0.001; 95% CI 0.00, 0.00].

**FIGURE 4:**
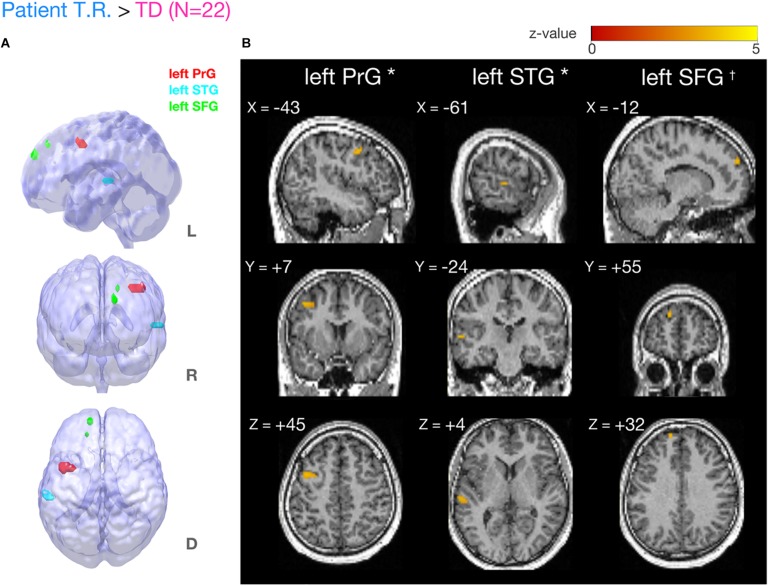
Brain regions more strongly activated in T.R. than in control subjects during the tactile temporal order judgment (TOJ) task. **(A,B)** Group level results after small volume correction (SVC; voxel level *p* < 0.001, uncorrected; cluster level *p*FWE < 0.05; TOJ > NJ) in the left precentral gyrus (PrG) (**A**, red), superior temporal gyrus (STG) (**A**, blue), and superior frontal gyrus (SFG) (**A**, green) with the contrast. Note that left SFG^†^ narrowly missed statistical significance after cluster correction (*p*FWE = 0.08). Results are mapped on volume rendered brain (**A**: L, lateral; R, rostral; D, dorsal views) and sections [**B:** x, sagittal; y, coronal; z, axial slices, Montreal Neurological Institute (MNI) coordinates] created from T.R.’s T1 image. FWE, Family Wise Error.

Our fMRI analysis focused on a few *a priori* anatomical brain regions as explained (see section “Introduction”). Comparisons of fMRI-BOLD activity (task contrast: TOJ > NJ) between T.R. and 22 TD controls using a modified *t*-test specifically designed for case studies (Crawford and Howell, 1998; Crawford et al., 2006) revealed that T.R.’s neural responses were significantly stronger in the left PrG and left STG (Figure 4; Table 2). Although the left SFG and right IFG showed greater activation in T.R. than in controls, it was not statistically significant after correcting for multiple comparisons within the volume of this ROI (*p*FWE = 0.08).

**TABLE 2:** Brain regions more strongly activated in patient T.R. than TD control subjects (*N* = 22) in the tactile TOJ task (TOJ > NJ contrast).

							**MNI Coordinates**		
**Cluster#**	**Size (voxel)**	***z*-value**	**pFWE**	**L/R**	**Region**	**BA**	***x***	***y***	***z***
1	45	4.01	0.014	L	Precentral Gyrus	6	−43	7	45
2	12	3.89	0.088	L	Superior Frontal Gyrus	9	−12	55	32
3	18	4.11	0.036	L	Superior Temporal Gyrus	22	−61	−24	4

*Results were initially thresholded at *p* < 0.001 (voxel level, uncorrected) and then cluster corrected at *p*FWE < 0.05. BA, Brodmann area; FWE, Family Wise Error; MNI, Montreal Neurological Institute (1 voxel = 2.2 × 2.2 × 3.2 mm^3^); NJ, numerosity judgment; TOJ, temporal order judgment.*

**FIGURE 5:**
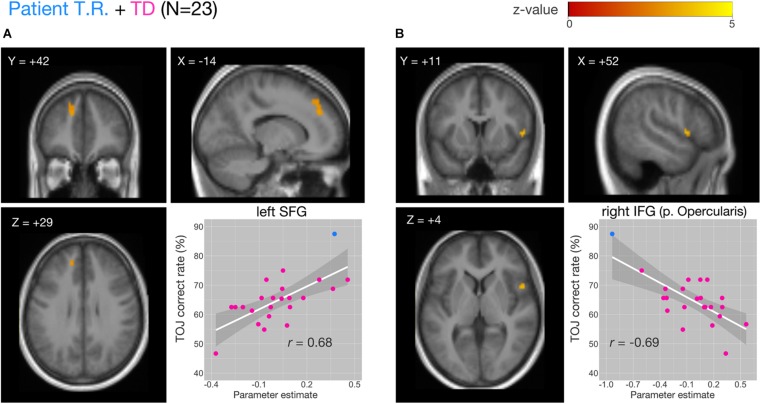
Correlations of functional MRI (fMRI) activation [temporal order judgment (TOJ) > numerosity judgment (NJ)] and correct TOJ performance rates in the left superior frontal gyrus (SFG) and the right inferior frontal gyrus (IFG) after small volume correction (SVC). The analyses were thresholded at voxel level *p* < 0.001, uncorrected and cluster corrected with *p*FWE < 0.05. Significant clusters were mapped on sections [x, sagittal; y, coronal; z, axial slices, Montreal Neurological Institute (MNI) coordinates] created from averaged, normalized, T1-weighted images of 23 subjects [the patient T.R. + 22 typically developing (TD) controls]. The correct TOJ rates are plotted against the extracted SPM parameter estimates from the identified clusters in left SFG (**A**, inset) and right IFG (**B**, inset) wherein filled circles show individual rates of T.R. (blue) and TD controls (magenta). Shaded regions represent 95% confidence intervals of the Pearson’s correlation coefficients.

**FIGURE 6:**
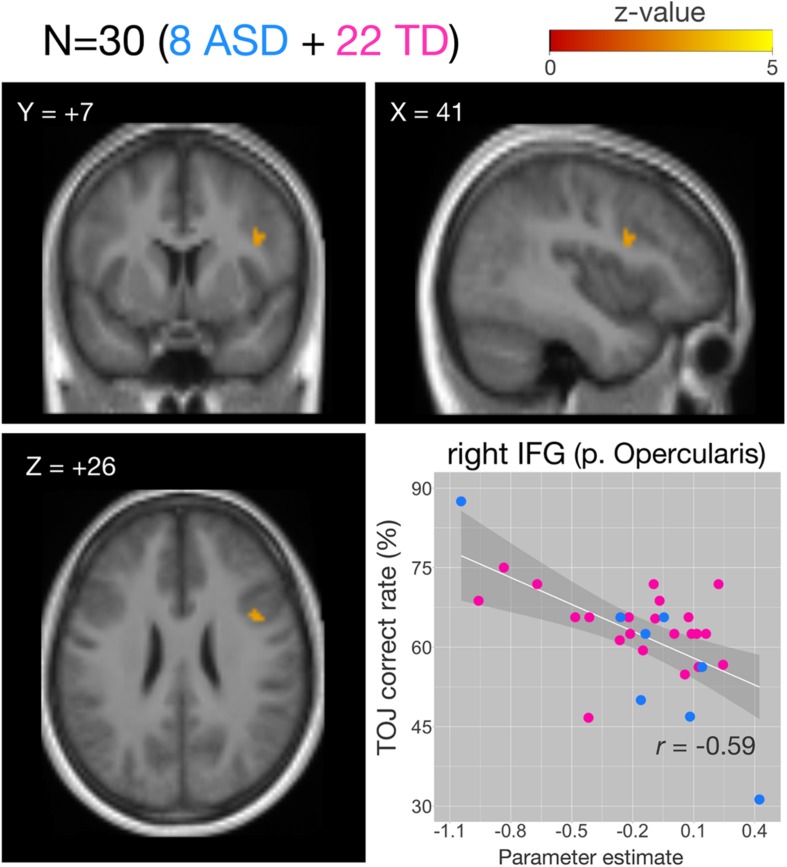
Correlations of functional MRI (fMRI) activation [temporal order judgment (TOJ) > numerosity judgment (NJ)] and correct TOJ performance rates in the right inferior frontal gyrus (IFG) after small volume correction (SVC). The analyses were thresholded at voxel level *p* < 0.001, uncorrected and cluster corrected with *p*FWE < 0.05. Significant clusters were mapped on sections [x, sagittal; y, coronal; z axial slices, Montreal Neurological Institute (MNI) coordinates] created from averaged, normalized, T1-weighted image of 30 subjects. The correct TOJ rates are plotted against the extracted SPM parameter estimates from the identified clusters in the right IFG wherein filled circles show individual rates of eight autism spectrum disorder (ASD) (blue) and 22 healthy controls (magenta). Shaded regions represent 95% confidence intervals of the Pearson’s correlation coefficients. FWE, Family Wise Error.

We examined the relationship between TOJ performance accuracy and brain activity within the same set of *a priori* ROIs. The rate of correct TOJ performances was correlated with BOLD signals (TOJ > NJ) for T.R. with 22 TD subjects. A significant positive correlation was found only in the left SFG (Figure 5A), whereas a negative correlation was evident in the opercular part of right IFG (Figure 5B; Table 3). To confirm that these brain regions assumed to modulate the accuracy of TOJ are common between TD and ASD, we performed the analysis of the same 22 TD with eight ASD subjects (including T.R.). Again, we found a significant negative correlation in the right IFG while did not find any correlation in the left SFG (Figure 6; Table 4). Although the region appears to be a more posterior part compared to the region shown in Figure 5B (i.e. the right IFG), note that those were anatomically identified as same regions in SPM Anatomy toolbox (Eickhoff et al., 2005).

**TABLE 3:** Activity of brain regions (TOJ > NJ contrast) showing significant positive (Cluster #1) and negative (Cluster #2) associations with correct tactile TOJ rates in 23 subjects (the patient + 22 TD controls).

							**MNI Coordinates**		
**Cluster#**	**Size (voxel)**	***z*-value**	**pFWE**	**L/R**	**Region**	**BA**	***x***	***y***	***z***
1	51	3.61	0.014	L	Superior frontal gyrus	9	−14	42	29
2	13	3.83	0.036	R	IFG (p. Opercularis)	44	52	11	4

*Results were initially thresholded at *p* < 0.001 (voxel level, uncorrected) and then cluster corrected at *p*FWE < 0.05. All other conventions are as in Table 2. BA, Brodmann area; FWE, family wise error; IFG, inferior frontal gyrus; MNI, Montreal Neurological Institute (1 voxel = 2.2^3^ × 2.2^3^ × 3.2 mm^3^); NJ, numerosity judgment; TD, typically developing; TOJ, temporal order judgment.*

**TABLE 4:** Activity of brain regions (TOJ > NJ contrast) showing significant negative (Cluster #1) associations with correct tactile TOJ rates in 30 subjects (the patient + 7 ASD + 22 TD controls).

							**MNI Coordinates**		
**Cluster#**	**Size (voxel)**	***z*-value**	**pFWE**	**L/R**	**Region**	**BA**	***x***	***y***	***z***
1	12	3.41	0.039	R	IFG (p. Opercularis)	44	41	7	26

*Results were initially thresholded at *p* < 0.001 (voxel level, uncorrected) and then cluster corrected at *p*FWE < 0.05. All other conventions are as in Table 2. ASD, autism spectrum disorder; BA, Brodmann area; FWE, Family Wise Error; IFG, inferior frontal gyrus; MNI, Montreal Neurological Institute (1 voxel = 2.2 × 2.2 × 3.2 mm^3^); NJ, numerosity judgment; TD, typically developing; TOJ, temporal order judgment.*

## Discussion

Although fMRI studies have reported neural correlates of sub-second timing processes during TOJ, so far, an interplay of facilitatory/inhibitory functions toward superior temporal resolution is less known. To our knowledge, patient T.R. studied here would be the first case of prominent sensory processing of short, sub-second timing. This offers insight into the underlying neural substrates of heightened sensory processing demonstrated by individuals with ASD. Thus far, studies have focused on the processing of local details in the spatial domain (O’Riordan et al., 2001; O’Riordan, 2004; Kaldy et al., 2016; Cheung et al., 2018). T.R.’s tactile temporal resolution of 6.49 ms studied here fell markedly outside the range of that reported in previous data from healthy subjects (Yamamoto and Kitazawa, 2001; Takahashi et al., 2013; Ide et al., 2019) (e.g. mean: 74 ms; range: 30–131 ms; Yamamoto and Kitazawa, 2001). Areas previously reported as TOJ-specific in healthy individuals showed stronger activations in PrG and STG in T.R. than those in controls. Of these, the opercular part of the right IFG was significantly associated with overall correct TOJ rate across all subjects (T.R. and controls). Especially, weaker brain activity in the right IFG was associated with facilitation of temporal resolution irrespective of diagnosis (i.e. ASDs and TD controls).

Lateralization to the left hemisphere has been reported earlier (Wittmann et al., 2004; Moser et al., 2009; Binder, 2015), and the left vPMC was found to be involved as a TOJ-related region (Miyazaki et al., 2016). This suggests that greater amplitudes of neural activity in left-lateralized regions may associate with increased efficiency for processing smaller intervals of tactile stimuli. However, it is difficult to conclude the contribution of TOJ-related regions in the processing of smaller intervals only in the left hemisphere because the present study did not test laterality itself. SFG involved in a modulatory role in the current study was shown to be involved especially in spatial WM system (Rowe et al., 2000; Johnson et al., 2003). Since successful TOJ performance necessitates mapping of somatosensory information to external space (Heed and Azañón, 2014; Binder, 2015), a more accurate spatial mapping of tactile inputs may have resulted in higher temporal resolution in T.R.

Most importantly, our correlation analysis including both TD and ASD demonstrated that weaker activation in the right IFG may be considerably essential for fine timing processes. Brain activity in that region has been reported to be increased in a short SOA condition of TOJ and was shown to associate with task difficulty (Lewandowska et al., 2010). Other fMRI studies have demonstrated that the right IFG is activated in response to task demands of motor inhibition (Konishi et al., 1998; Garavan et al., 1999; Konishi et al., 1999; Menon et al., 2001). In agreement with that, patients with BA 44 damage had difficulty in rapidly inhibiting motor responses in a stop-signal task (Aron et al., 2003). Owing to the strong connection between the motor and somatosensory areas (Catani, 2017), it is possible that disinhibition of the somatosensory areas by decreased activity in the right IFG resulted in better temporal resolution of tactile inputs as evident in our results. However, we need to be cautious in interpreting the results as the sample size in our study was small, whereas relatively larger sample size is recommended to obtain reliable and stable result in correlation analysis (Schönbrodt and Perugini, 2013).

In contrast to a previous report that neural activities in the bilateral IFG increased depending on task difficulty for TD individuals (Lewandowska et al., 2010), our result demonstrated that subjects with higher temporal resolution tended to show lower neural responses in the right IFG during TOJ. Another report suggested that synchronous neural activities in alpha and beta frequencies associated with the right IFG and primary somatosensory area were observed when performing a tactile detection task with inhibitory task-irrelevant sensory stimuli (Sacchet et al., 2015). We speculate that the right IFG with synchronous neural activation along with the primary somatosensory area is crucial for performing difficult perceptual/cognitive tasks and not limited to exhibiting higher temporal resolution. Since the important role of right IFG is response inhibition to stop-signals (Aron et al., 2003; Depue et al., 2010; Bodmer and Beste, 2017), disinhibition of sensory inputs from the primary somatosensory area might result in the extraordinary high temporal resolution. We need further studies to examine how the right IFG functions when subjects are required to perform perceptual/cognitive tasks without having to inhibit any distracting stimuli.

## Conclusion

Brain regions of mainly the left hemisphere hypothesized to contribute in superior temporal resolution were strongly activated during TOJ in the patient T.R. who manifested prominent sensory processing. Right IFG played a modulatory role in regulating temporal resolution generally in ASD and healthy individuals. Specifically, the result of correlation analysis that included ASD and healthy subjects demonstrated a prominent role of the right IFG in modulating the neural basis of time processing. These findings add valuable insights into the underlying neural basis of fine scales of time processing in the brain.

## Data Availability Statement

The data that support the findings of this study are available from the corresponding author (MI) upon reasonable request. Readers are welcome to comment on the online version of the manuscript. Correspondence and requests for materials should be addressed to author MI (ide-masakazu@rehab.go.jp).

## Ethics Statement

The studies involving human participants were reviewed and approved by the Ethics committee of the National Rehabilitation Center for Persons with Disabilities. Written informed consent to participate in this study was provided by the participants’ legal guardian/next of kin. Written informed consent was obtained from the individual(s) for the publication of any potentially identifiable images or data included in this manuscript.

## Author Contributions

MI, TA, MC, AY, and MW planned the study. MI, TA, MC, AY, and YU conducted the experiments. MI analyzed the behavioral data, and TA and MC analyzed the MRI data. MI, TA, MC, RF, and MW interpreted the results. MI, TA, and MC wrote the manuscript. All authors read the manuscript, gave relevant inputs, and approved the final version of the same.

## Conflict of Interest

The authors declare that the research was conducted in the absence of any commercial or financial relationships that could be construed as a potential conflict of interest.

## Footnotes

1 http://www.fil.ion.ucl.ac.uk/spm

2 www.mricro.com

3 http://medicine.yale.edu/bioimaging/suite/mni2tal/

4 http://www.talairach.org/client.html

5 http://imaging.mrc-cbu.cam.ac.uk/imaging/MniTalairach
