# Supplementary material for: Neural Basis of Extremely High Temporal Sensitivity: Insights From a Patient With Autism
Source: Front Neurosci. 2020 Apr 30;14:340. doi: 10.3389/fnins.2020.00340 (PMC7203484; doi:10.3389/fnins.2020.00340)
Supplement: Supplementary file 1 [file Data_Sheet_1.pdf]

Supplementary materials

Table of Contents

I. Overlap of subjects for TOJ tasks across different modalities. – Supplementary Table 1

II. Task performances of T.R. in same SOA conditions – Supplementary Figure 1

<u>Supplementary Table 1</u>

Supplementary Table 1. Overlap of typically developing subjects for TOJ tasks across different modalities.

The numbers denote the counts that the subjects who performed the tasks with the combinations of modalities.

	Bilateral Tactile	Auditory	Visual	Unilateral Tactile
**Bilateral Tactile**		3	7	4
**Auditory**	3		2	5
**Visual**	7	2		2
**Unilateral Tactile**	4	5	2	

<u>**Supplementary Figure 1**</u>

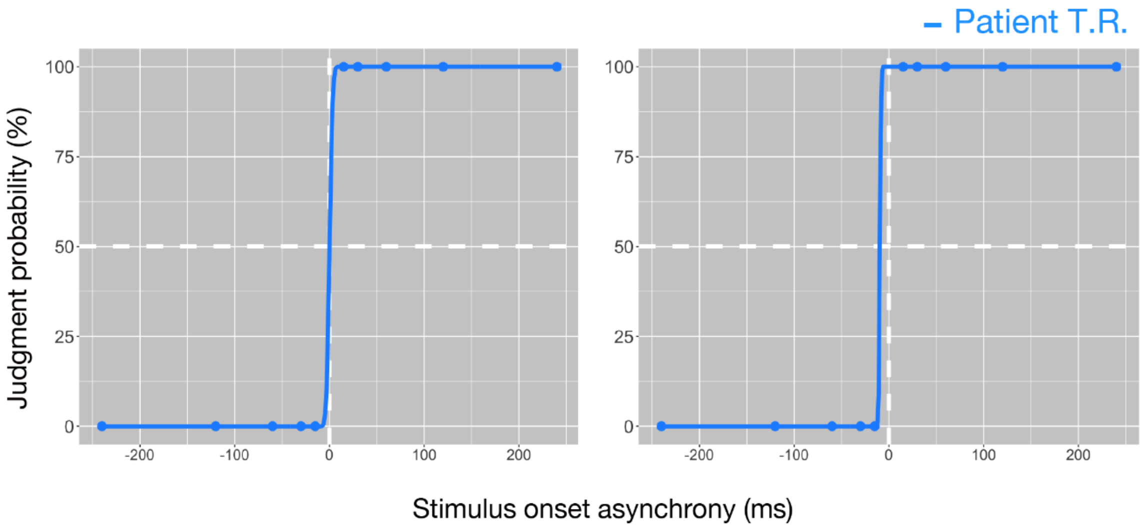

Supplementary Figure 1. Temporal resolutions in TOJ task of 40 Hz (left) and 200 Hz (right) vibrotactile stimuli in patient T.R.

The judgment probability (y-axis) that stimuli delivered to one side was earlier than the (right) other is plotted against the stimulus onset asynchrony (x-axis) in 40 Hz (left) and 200 Hz vibration conditions. Positive values along x-axis indicate that the right side was stimulated first. Each filled circle represents judgment probabilities calculated from 20 responses; curve represents the model fit to the data (see the Material and Methods section).
